# Prognostic impact of tumor mutation burden and the mutation in *KIAA1211* in small cell lung cancer

**DOI:** 10.1186/s12931-019-1205-9

**Published:** 2019-11-07

**Authors:** Mengting Zhou, Jun Fan, Zhenyu Li, Pindong Li, Yajie Sun, Yuhui Yang, Xiaoshu Zhou, Jing Wang, Ye Wang, Huiwei Qi, Weijing Cai, Xiaofang Dai, Fred R. Hirsch

**Affiliations:** 1 Cancer Center, Union Hospital, Tongji Medical College, Huazhong University of Science and Technology, 1277 JieFang Avenue, Wuhan, 430022 People’s Republic of China; 2 Department of Pathology, Union Hospital, Tongji Medical College, Huazhong University of Science and Technology, Wuhan, 430022 People’s Republic of China; 3 Shanghai Tongshu Biotechnology Co., Ltd, Shanghai, 200120 People’s Republic of China; 4 Center for Thoracic Oncology, Tisch Cancer Center Mount Sinai Health System; Icahn School of Medicine at Mount Sinai, New York, USA

**Keywords:** Small cell lung cancer, Genomic alterations, Tumor mutation burden, *KIAA1211*, Overall survival, Progression-free survival

## Abstract

**Background:**

Small cell lung cancer (SCLC) is a highly aggressive lung cancer subtype with poor survival and limited treatment options. Sequencing results have revealed gene mutations associated with SCLC, however, the correlation between the genomic alterations and clinical prognosis of SCLC is yet unclear.

**Methods:**

Targeted next-generation sequencing of 62 cancer related genes was performed on 53 SCLC samples. The correlations between clinical outcomes and genomic alterations were analyzed.

**Results:**

38/62 (61.3%) candidate genes harbored some alterations, while all the SCLC samples carried at least 3 gene mutations. The most common nonsynonymous mutations included *ERBB2* (95.9%), *CREBBP* (95.9%), and *TP53* (77.6%). The median nonsynonymous tumor mutation burden (TMB) was 21.7 mutations/Mb (rang, 9.3–55.9). High TMB (> 21 mutations/Mb) was good prognostic factor in overall survival (OS) (21.7 vs. 10.4 months, *P* = 0.012). Multivariate analysis showed that high TMB was an independent prognostic factor. The overall survival (OS) of patients carrying *KIAA1211* mutation was significantly longer than those with wild-type *KIAA1211* (*P* < 0.001).

**Conclusions:**

The current study highlights the potential role of genomic alterations for the prognosis of SCLC. Higher TMB was associated with a better prognosis, and *KIAA1211* might be a good prognostic factor in SCLC.

## Background

Lung cancer is the leading cause of cancer deaths in both women and men in the China and throughout the world [1]. Small-cell lung cancer (SCLC) accounts for approximately 10–15% of all lung cancers. It is a highly aggressive malignancy frequently presenting with metastases at the time of diagnosis [2]. Most patients respond to chemotherapy, unfortunately, the majority suffer disease recurrence or progression sooner rather than later. Treatment options have remained unchanged for the past three decades. Furthermore, until now, the most reproducible prognostic factor is stage of the disease and molecular biomarkers are still lacking [3]. Therefore, to better understand the clinical outcomes, it is essential to explore the genetic alterations and identify prognostic biomarkers.

The genetic mutational landscape of SCLC is complex and heterogeneous, however, the most common genetic alterations include inactivation of tumor suppressor genes *TP53* and *RB1*, copy number gains in *MYC* family members, enzymes involved in chromatin remodeling, and kinases signaling pathways [4, 5]. No targeted drug has showed significant anti-tumor activity in SCLC until now. Recently, immune checkpoint inhibitors have shown efficacy in SCLC with PD-1 inhibition. Pembrolizumab demonstrated promising antitumor activity in SCLC with an objective response rate (ORR) of 33% [6], while nivolumab had an ORR of 10% as monotherapy or 19–23% in combination with ipilimumab in patients with relapsed SCLC [7]. The combination of atezolizumab and chemotherapy (platinum + etoposide) was recently approved by the US Food and Drug Agency (FDA) and led to a new treatment paradigm for extensive SCLC [8]. Besides programmed death ligand 1 (PD-L1) expression, tumor mutation burden (TMB) is regarded as a biomarker of the efficacy of programmed death 1 (PD-1) inhibitors in various cancers. Thus, a deeper understanding of the driver alterations in SCLC, and an understanding of those patients likely to respond to immune checkpoint blockade should improve patient outcome. A few seminal genomic studies have been conducted [9–11], and the genomic features have been correlated with the clinical outcome. However, these studies were evaluated patients with surgically resected tumors, and there were few kinds of research on Asian populations. Moreover, the relation between TMB and prognosis in SCLC is still unclear.

Here we employed selected 62 exome sequencing in SCLC and analyzed the genomic profiling and the potential association with the clinical outcomes.

## Methods

### Patients and samples

From May 2014 to January 2017, a total of 53 SCLCs and matched normal lung formalin-fixed and paraffin-embedded (FFPE) tissue samples were obtained from Wuhan Union Hospital, China. All clinicopathological data were retrospectively collected. The stage of SCLC were categorized by the older Veterans Administration Lung Study Group’s 2-stage classification scheme [12], which classified into limited-stage (LS) and extensive-stage (ES).

### DNA extraction

We performed DNA extraction from serial thick sections cut from tumor tissue samples and control sections. The invasive tumor content was estimated by pathologists, to ensure more than 50% of cells were tumor cells. The DNA was isolated from the FFPE and blood samples using the DNeasy Blood and Tissue Kit (69,504, QIAGEN, Venlo, Netherlands).

### Next-generation sequencing

We performed targeted sequencing of 62 cancer related genes using an amplicon based sequencing panel of Ion AmpliSeq™ (Life Technologies, Carlsbad, USA), and then generated sequence data using Ion Proton™ System (Life Technologies, Carlsbad, USA).

### Statistical analysis

Fisher’s exact test was used to compare the frequency data between two groups. Survival data were calculated using the Kaplan–Meier method and survival curves were compared with the log-rank test. The variables putatively associated with survival were analyzed with the Cox proportional hazards test. All tests were bilateral, with *P* < 0.05 indicating significant statistical difference. Statistical analysis was carried out by the statistical software package SPSS 22.0 (IBM Corp., Somers, NY, USA).

## Results

### Clinicopathological characteristics of SCLC patients

The present study enrolled a total of 53 patients: 49 males and 4 females. The median age of the patients was 60 years (range, 57–66 years). The Eastern Cooperative Oncology Group Performance Status (ECOG PS) of 46 patients was ≤1, and that of 7 cases was =2; 24 cases were LS-SCLC and 29 were ES-SCLC; 42 cases presented a smoking history. Additionally, 64.2% of the patients did not have a history of chronic diseases.

All patients received first-line chemotherapy; 50 (94.3%) received platinum-based doublet chemotherapy, 2 underwent irinotecan monotherapy, and 1 had etoposide monotherapy. A total of 35 patients (66.0%) received chest radiotherapy; of these, 9 (25.7%) underwent concurrent chemoradiotherapy, while the remaining received sequential radiotherapy (Table 1).

**Table 1:** Clinical features of SCLC patients

Characteristics	All patients (*N* = 53) (%)
Age	60
< 60	25 (47.2)
≥ 60	28 (52.8)
Gender	
Male	4 9 (92.5)
Female	4 (7.5)
Clinical Stages	
Limited stage	24 (45.3)
Extensive stage	29 (54.7)
Smoking	
Never smoker	11 (20.8)
Smoker	42 (79.2)
ECOG PS Score	
0	5 (9.4)
1	41 (77.4)
2	7 (13.2)
Past Medical History, NO (%)	
Cardiopathy	1 (1.9)
Diabetes	2 (3.8)
Hypertension	11 (20.8)
Hypertension & Diabetes	2 (3.8)
Respiratory Disease	3 (5.7)
None	34 (64.2)
Radiotherapy	
Yes	35 (66.0)
No	18 (34.0)

### Distribution of gene mutations

**Fig. 1:**
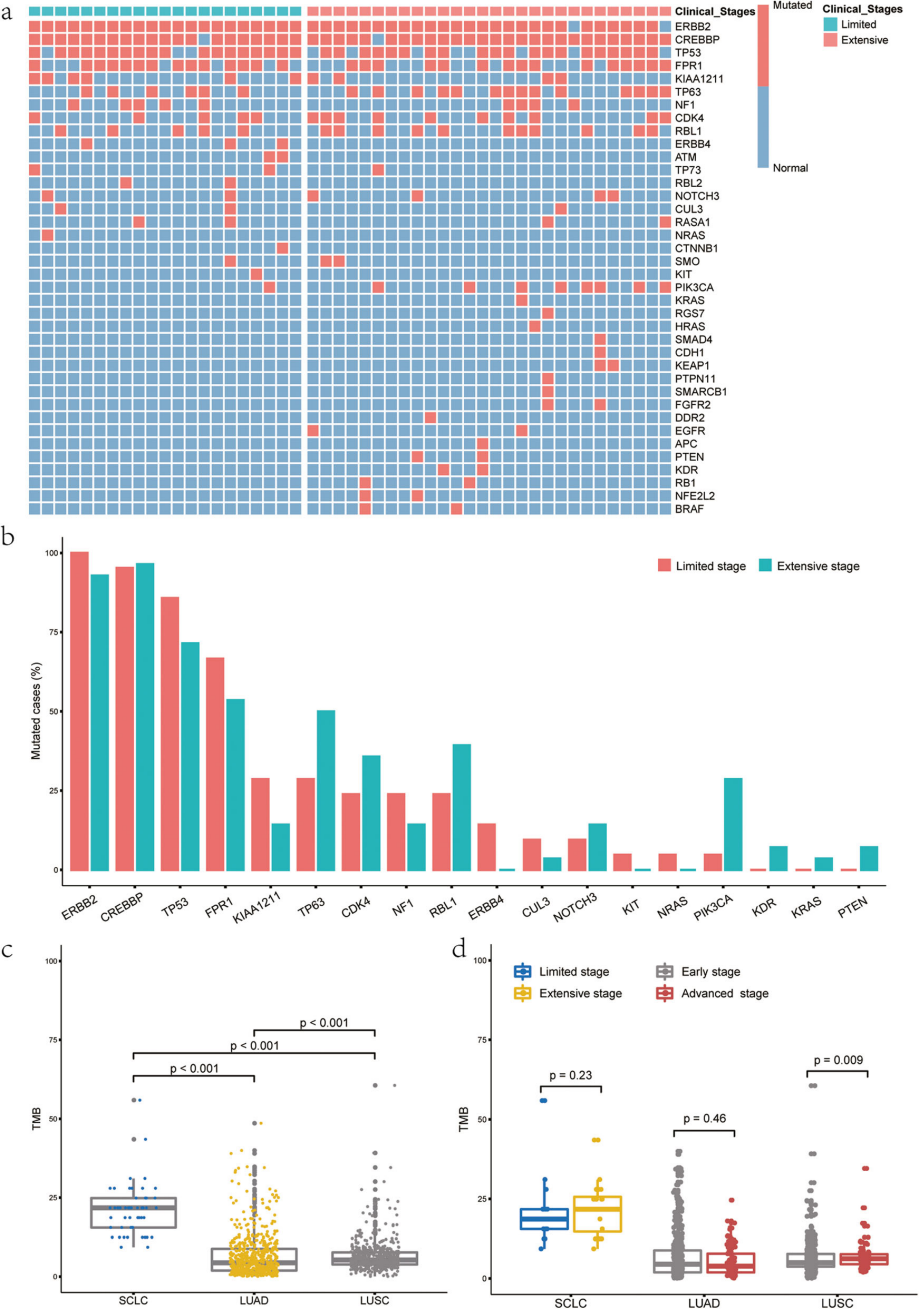
Genomic alterations and TMB in SCLC. **a**-**b** Genomic alterations in the extensive stage SCLC and the limited stage SCLC; **c** TMB in SCLC versus lung adenocarcinoma (LUAD) and lung squamous carcinoma (LUSC) from TCGA; **d** TMB in extensive stage SCLC versus limited stage SCLC and TMB in early stage LUAD/LUSC versus advanced stage LUAD/LUSC

A total of 62 candidate genes were sequenced from 49/53 SCLC samples. Consequently, alterations were detected in 38 genes. All the SCLC samples carried a minimum of 3 mutations. The most common nonsynonymous mutations occurred in *ERBB2* (95.9%), *CREBBP* (95.9%), and *TP53* (77.6%) (Fig. 1a). We also analyzed the distribution of variants. A total of 156 nonsynonymous variants were identified, The most common nonsynonymous variants were *ERBB2*.p.L755 M (95.9%) and *CREBBP*.p.V1780 M (91.8%), which were primarily concentrated at one variant (> 90%)(Additional file 1: Figure S1). The variants of *ERBB4*, *KIT* and *NRAS* were only observed in LS-SCLC, while the variants of *KDR*, *KRAS* and *PTEN* were only detected in ES-SCLC. There was no significant difference in the mutation rates of the above variants between different stages (Fig. 1b) because of the small number of mutations.

### The relationship between TMB and prognosis

**Fig. 2:**
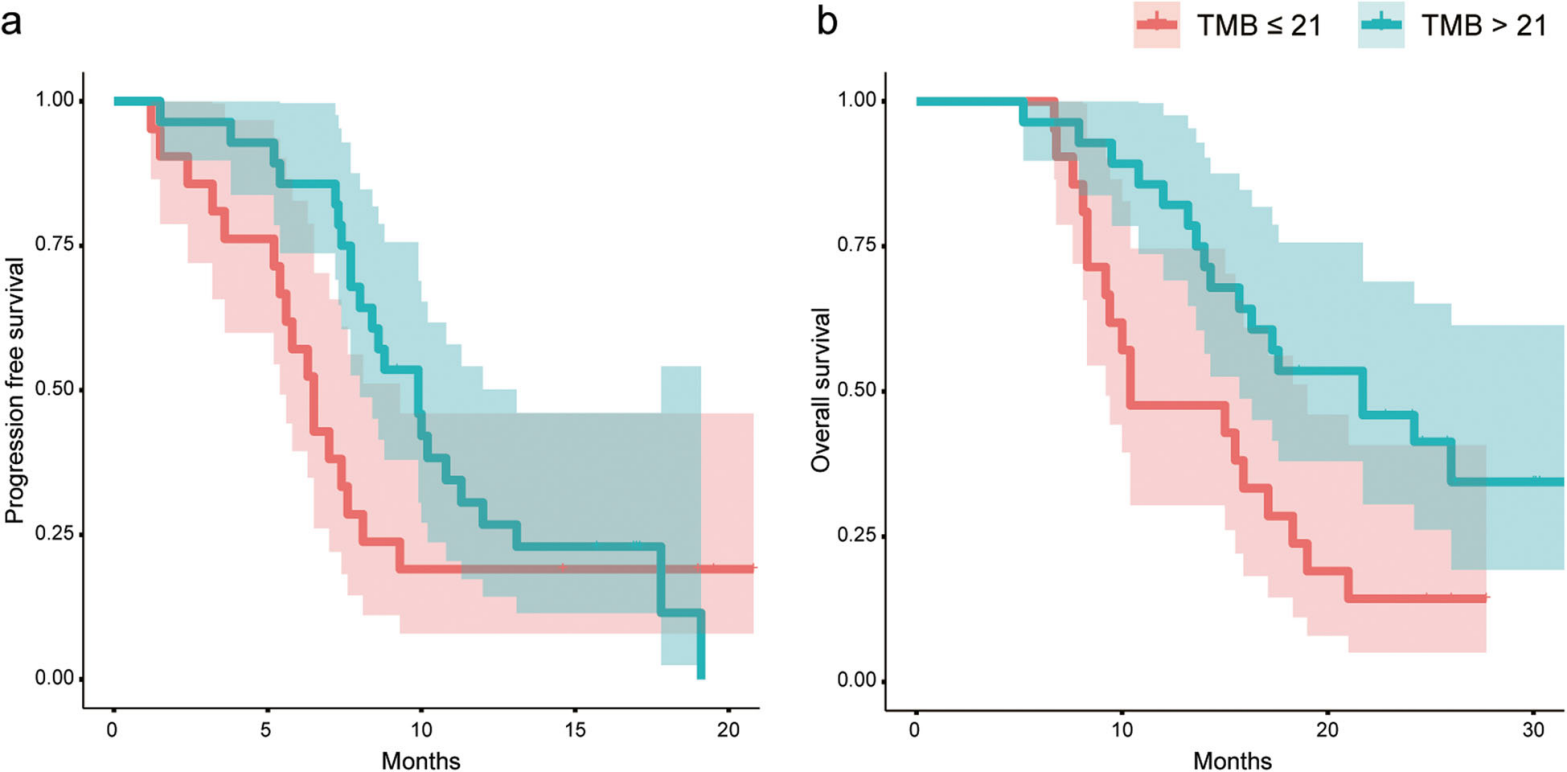
Kaplan–Meier analysis of high TMB and low TMB. **a**. Progression free survival; **b**. Overall survival

The median number of nonsynonymous variants carried by each sample was 7 (range, 3–18). Nonsynonymous TMB was estimated and analyses were performed to assess the effect of TMB on prognosis. The genomic coverage of the panel is 0.322 Mb. The median TMB was 21.7 mutations/Mb (rang, 9.3–55.9) (Fig. 1c). We defined 21 mutations/Mb as the cut-off value, TMB > 21 mutations/Mb as high and TMB ≤ 21 mutations/Mb as low. Univariate analysis showed longer progression free survival (PFS) with high TMB than low TMB (9.9 vs. 6.5 months) but without statistically significance (*P* = 0.16) (Fig. 2a), and overall survival (OS) was significantly longer in high TMB than low TMB (21.7 vs. 10.4 months, *P* = 0.012) (Fig. 2b). Multivariate analysis showed that high TMB was an independent prognostic factor in PFS (*P* = 0.004) and OS (*P* < 0.001) with adjustment for age, sex, smoking status, ECOG PS, and stage (Table 2). The results were consistent across the subgroups of ES-SCLC (PFS: *P* = 0.16, OS: *P* < 0.001) (Additional file 2: Figure S2).

**Table 2:** Multivariate analysis of PFS and OS

	PFS			OS		
	HR	95%CI	*P* value	HR	95%CI	*P* value
Age > 60	1.63	0.81–3.26	0.17	2.23	1.07–4.65	0.03
Female	1.31	0.28–6.16	0.73	2.32	0.37–14.36	0.36
Smoking	1.49	0.53–4.22	0.45	1.96	0.61–6.31	0.26
PS Score 0–1	0.32	0.09–1.10	0.07	1.07	0.23–4.89	0.93
Extensive Stage	2.03	0.91–4.57	0.08	2.70	1.12–6.47	0.03
TMB > 21 mutations/Mb	0.27	0.11–0.66	0.004	0.17	0.06–0.44	0.0003

In addition, compared with lung cancer data from the Cancer Genome Atlas (TCGA), the estimates of TMB in SCLC was much higher than TMB in lung adenocarcinoma (LUAD) and lung squamous carcinoma (LUSC) (*P* < 0.001) (Fig. 1c). But no significant difference in TMB between LS-SCLC and ES-SCLC was observed (Fig. 1d).

### Effects of genetic alterations on progression-free survival (PFS)

**Fig. 3:**
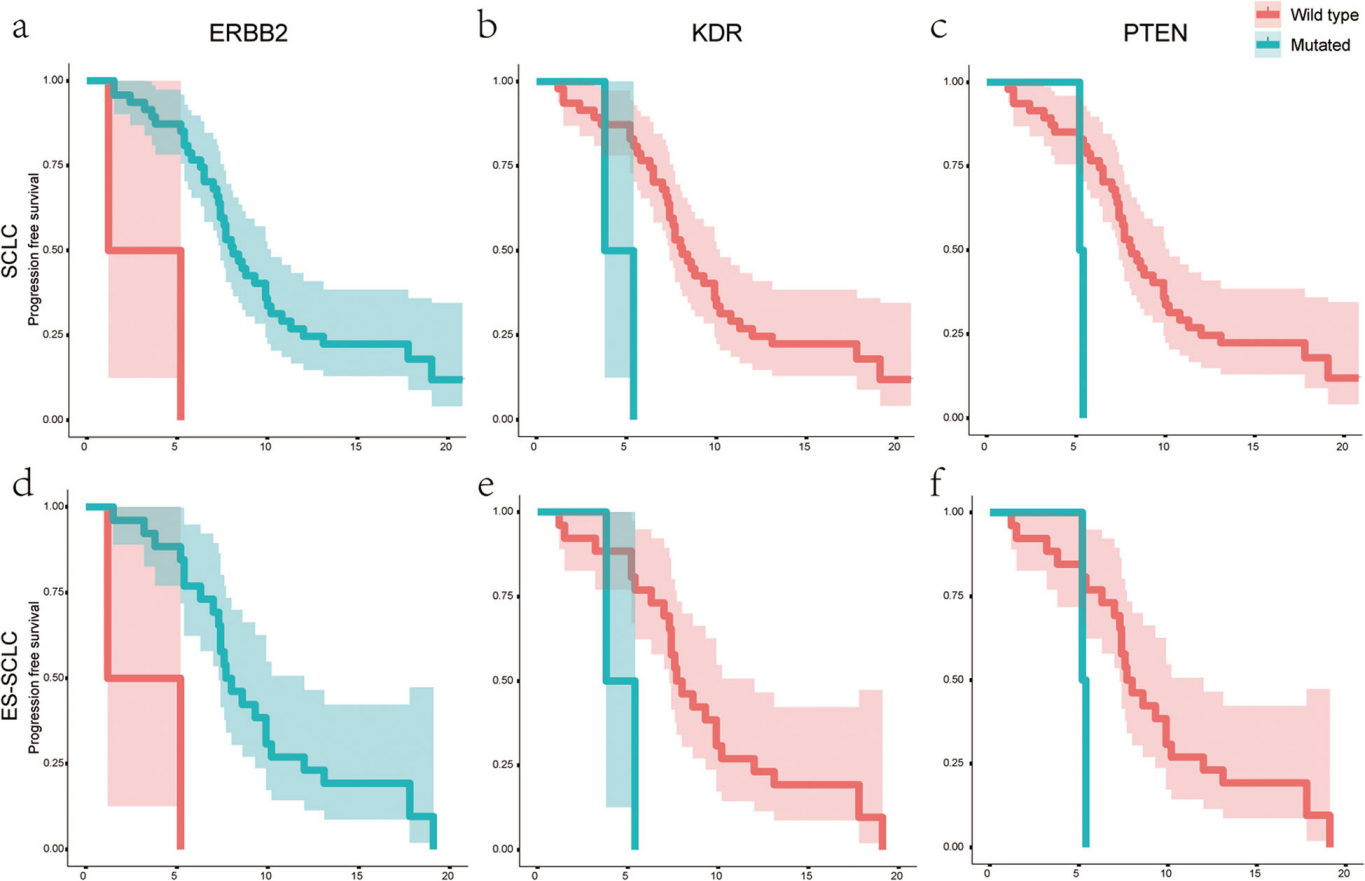
The univariate analysis of *ERBB2*, *KDR*, and *PTEN*. **a**-**c**. In total SCLC; **d**-**f**. In the extensive stage SCLC (ES-SCLC)

All SCLC patients were administered first-line chemotherapy. According to the evaluation of first-line treatment, 2 patients (4.1%) showed complete response (CR), 37 (75.5%) showed partial response (PR), 8 (16.3%) exhibited stable disease (SD), and 2 (4.1%) presented progression disease (PD). The median PFS was 8 months (range, 1.2–20.8 months). Univariate analysis revealed that patients with *ERBB2*mutations had a significantly prolonged PFS as compared to those with wild-type *ERBB2* (*P* < 0.001) (Fig. 3a, Additional file 3: Table S1). On the contrary, patients with *KDR* or *PTEN* mutations had a significantly reduced PFS as compared to those with wild-type *KDR* (*P* = 0.01) or *PTEN* (*P* = 0.017) (Fig. 3b, c, Additional file 3: Table S1). The results in ES-SCLC were similar (Fig. 3d, e, f). It is worth noting that *KDR* and *PTEN* mutations were only detected in ES-SCLC. However, there were only two samples with *KDR* or *PTEN* mutation, and only two samples did not carry *ERBB2* mutation, the above comparisons are meaningless. Thus, there were no significant correlation between mutations and PFS.

Among the 156 missense variants, 7 were analyzed after excluding those with low frequencies. Univariate analysis revealed that there was a significant correlation between *ERBB2*.p.L755 M variant and PFS (Additional file 3: Table S2). However, the *ERBB2* mutation detected in our study was concentrated at *ERBB2*.p.L755 M variant, it is meaningless to conduct the further analysis.

### Effects of genomic alterations on overall survival (OS)

**Fig. 4:**
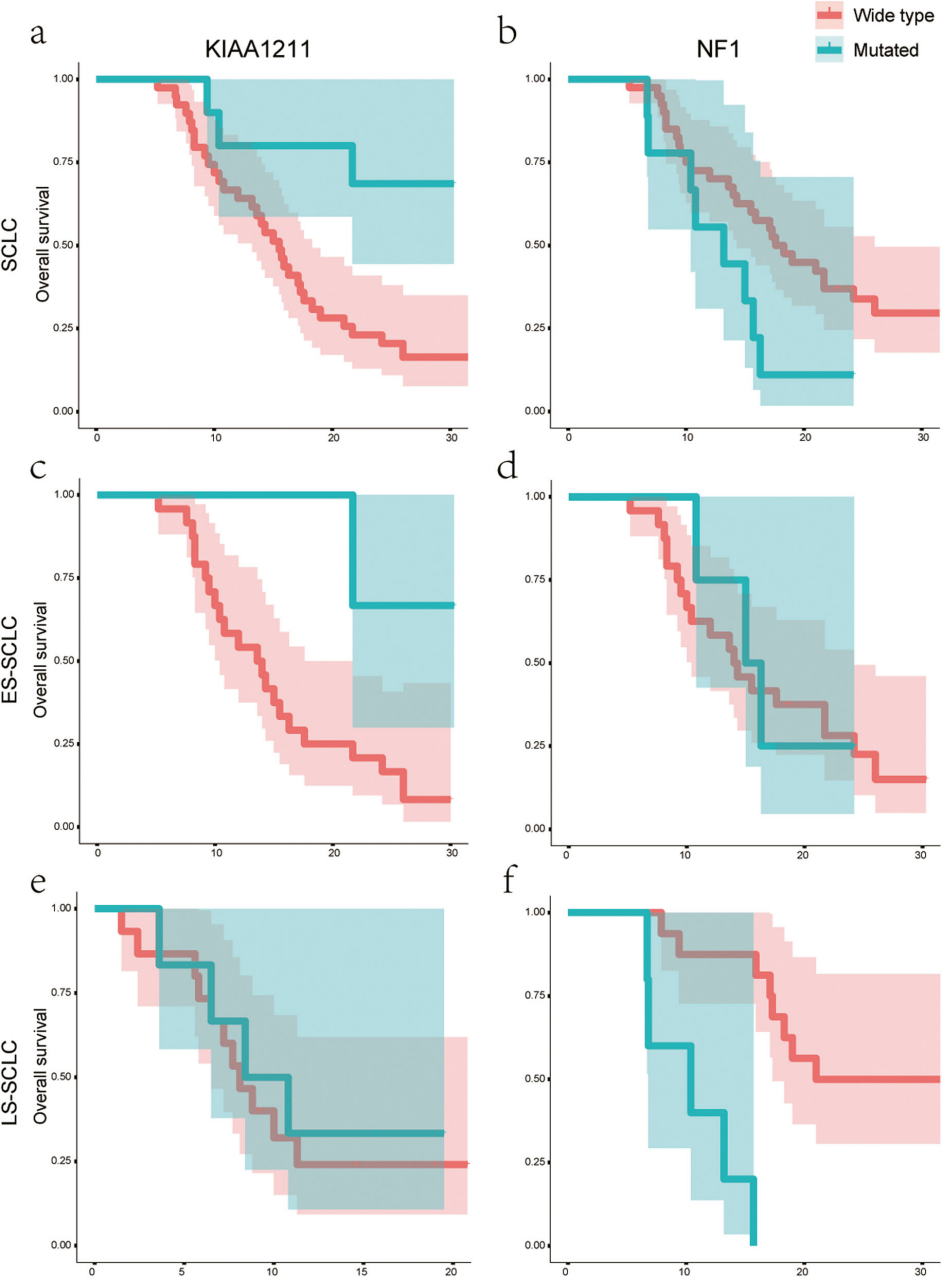
The univariate analysis of *KIAA1211* and *NF1*. **a**-**b**. In total SCLC; **c**-**d**. In the extensive stage SCLC (ES-SCLC); **e**-**f**. In the limited stage SCLC (LS-SCLC)

The average follow-up was for 16 months, and the median OS was 16.3 months (range, 5.2–32.5 months). Univariate analysis showed that patients with mutant *KIAA1211* had a longer OS than those with wild-type *KIAA1211* (*P* = 0.009) (Fig. 4a) (Additional file 3: Table S3). The results were similar in ES-SCLC (*P* = 0.032) but not in LS-SCLC (*P* = 0.19) (Fig. 4c, e). However, for patients with *NF1* mutations in LS-SCLC subgroup, their OS was significantly shorter than those with wild-type *NF1* (10.4 vs. 21 months, *P* < 0.001) (Fig. 4f). Similar results were not observed in either SCLC (*P* = 0.05) (Fig. 4b) or ES-SCLC (*P* = 0.78) (Fig. 4d) (Additional file 3: Table S3).

**Fig. 5:**
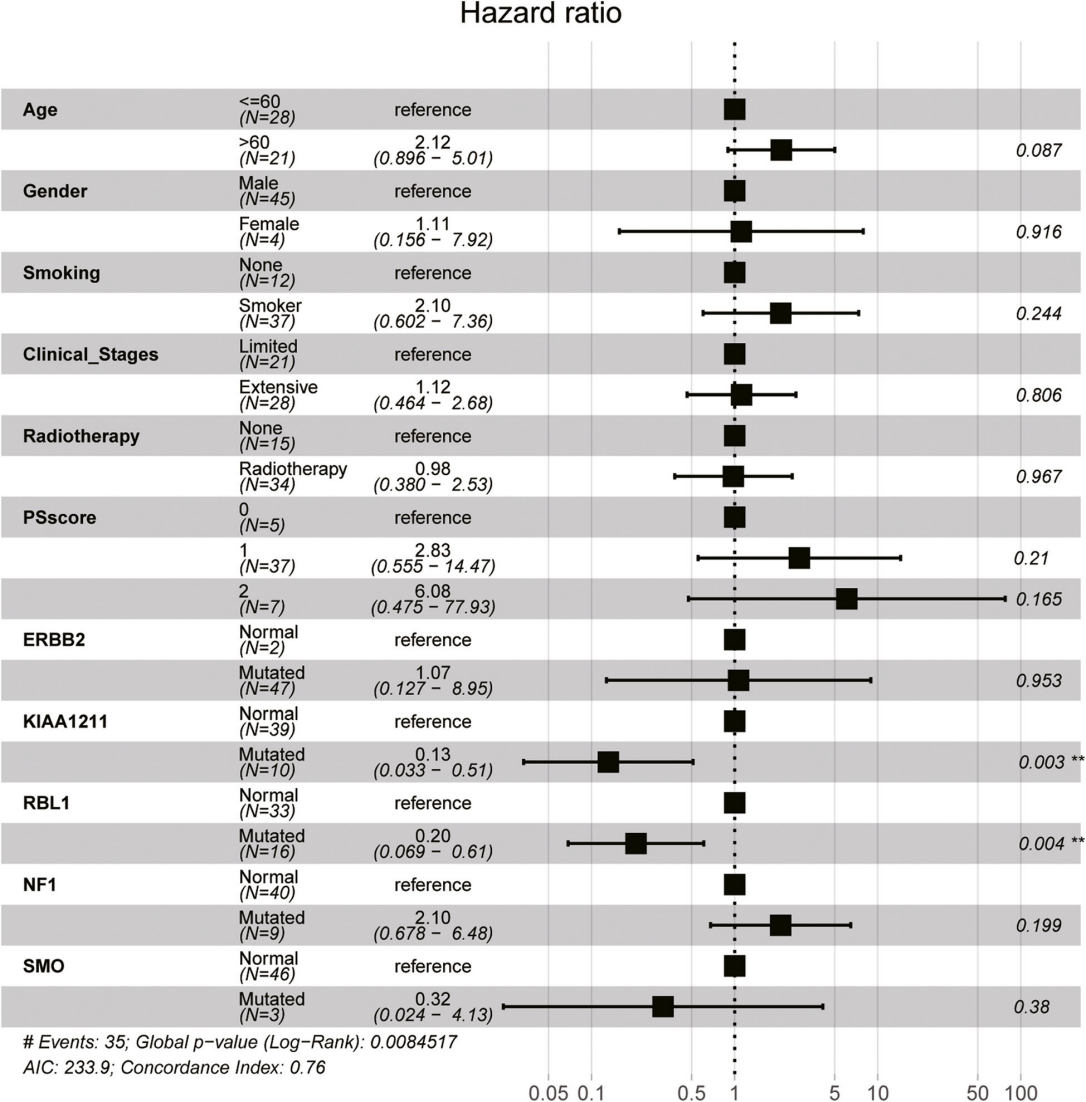
Multivariate analysis between mutations and overall survival

We established a Cox regression model using age, sex, smoking status, clinical stages, PS score, and 5 mutations as covariates for adjustment. The results demonstrated that the OS of patients carrying mutant *KIAA1211* was significantly longer than those with wild-type *KIAA1211* (*P* < 0.001) (Fig. 5), suggesting that *KIAA1211* mutation predicts a positive factor for SCLC prognosis. However, there was no significant correlation between variants and OS (Additional file 3: Table S4).

## Discussion

SCLC differs from other lung cancers irrespective of the pathological, molecular, or clinical manifestations. For nearly 30 years, no significant progress or effective targeted therapy for SCLC has been established, leading to still a poor prognosis [13]. Therefore, selection of an appropriate biomarker to assess the disease severity, monitor tumor progression, and evaluate the response to therapy is indispensable. Targeted therapy and immunotherapy aiming at specific genes improved the survival of SCLC patients; thus, identifying the genomic alterations of the patient and the effects of targeted genes on patient prognosis should be elucidated. Identifying and determining the biomarkers associated with SCLC prognosis would make a more careful assessment and classification of the SCLC population and could eventually also define subgroups of patients suitable for targeted therapies, which could improve the treatment outcome for defined subtypes and life quality for SCLC patients.

Gene sequencing-based diagnosis and treatment has been widely used among NSCLC patients, which improves the patient prognosis. In 2015, Thomas et al. [11], together with other groups, carried out a genome-wide sequencing of SCLC on the largest sample size to date. The study verified the results of previous genomic studies, such as common inactivation of *TP53* and *RB1* for SCLC, and also confirmed the disruption of other genes and signaling pathways, such as the *TP73* and *Notch* signaling pathway [14]. In SCLC, the inactivation of *TP53* and *RB1* accounted for 75–90% and 60–90%, respectively [15], which are the initial events in SCLC [11, 16, 17]. The above study also found that 13% of the SCLC samples carried *TP73* mutations or rearrangements. This phenomenon inhibited the function of wild-type *TP53*, which might be a potential target for the treatment of SCLC. Also, 25% of the SCLC samples were found to harbor the inactivated *Notch* gene, and animal studies confirmed that the *Notch* family exerts a tumor suppressive function and regulates the neuroendocrine differentiation of SCLC. SCLC has not been considered a homogenous tumor based on morphology. Tumor heterogeneity has been recognized many years ago: Mixed SCLC- Large cell tumors [14]. Some small sample studies also revealed tumor heterogeneity regarding the genomic analysis of SCLC. In the current study, mutations in *TP53*, *ERBB2*, and *CREBBP* were common with > 77% frequency. Moreover, we also detected mutations in *NOTCH3* and *TP73*, which occupied 12.2 and 6.1%, respectively, and other previously reported mutations such as *KIAA1211*, *RGS7*, and *FPR1* were also detected. Nevertheless, considerable differences were detected in the frequencies of significantly gene mutations in different studies, and the inconsistency might be attributed to the source of samples as well as different ethnicity of the patients.

Another study pointed out that the gene mutations and the total number of mutations were not associated with the OS or other clinical features of SCLC [18]. However, according to our findings, the mutations in *KIAA1211* prolonged the OS of patients, whereas mutations in *NF1* exhibit an opposite effect in LS-SCLC subgroup. *KIAA1211* was identified by the Kazusa cDNA project with uncharacterized biological functions [19]. Recently, *KIAA1211* was reported to transcriptionally upregulated in breast cancer [20]. While *KIAA1211* was frequently mutated or transcriptionally downregulated in colorectal cancer, furthermore, *KIAA1211* was demonstrated to act as a tumor suppressor through the maintaining of epithelial cell integrity [21]. In a comprehensive genomic analysis of somatic genome alterations in SCLC, *KIAA1211* was revealed to be a significantly mutated gene with a ranking of third following *TP53* and *RB1*, and it seems to involve the tumor pathogenesis [11]. However, *KIAA1211* was a newly discovered mutation in SCLC, its functional role in SCLC needs further investigation. In this study, we also observed a correlation between *ERBB2* mutation and PFS, but it lacks clinical significance due to the high occurrence rate of *ERBB2* mutation in SCLC. Furthermore, patients with higher TMB had a markedly prolonged OS, which indicated a better prognosis. Our results are similar to the previous results reported by Roszik et al. that patients with higher TMB had better clinical efficacy and prognosis in NSCLC [22]. However, in surgically treated NSCLC, high TMB is a poor prognostic factor [23]. The controversial results suggested that the validation of correlations of TMB with survival is needed.

Although molecular targeted therapy have not yet proven effective in SCLC, we detected some well-known oncogenic driver mutations including *PIK3CA* (9/49) [24], *KIT* (1/49) [25], and *BRAF* (2/49) [26], which suggested opportunities for more targeted therapeutic approaches.

Based on tumor characteristics, high-throughput sequencing of small panels underwent bioinformatics analysis. As a result, the mutation could be interpreted easily and rapidly, which reduces the economic burden of patients. This technique has many advantages, such as high targeting ability and cost-efficiency, in clinical practice. Hence, based on the previous studies, we established a panel of 62 genes closely associated with SCLC, sequenced the tumor samples from 53 SCLC patients, and analyzed the alterations of genes and the correlation with disease prognosis.

Nevertheless, the current study has several limitations. First, it is a retrospective study with a relatively small sample size, such that except for some common mutations, the others were low-frequency mutations, which could significantly affect the subsequent survival analysis. Second, because of the small panel, the estimates of TMB was higher in this study than TMB in the previous studies [27, 28]. In addition, we only detected two *RB1* mutations: *RB1*.p.R334STOP and *RB1*.p.R579QfsSTOP29. Several mutations in *RB1* occurred at exon-intron junctions, which caused protein-damaging splice events as confirmed by transcriptome sequencing. This phenomenon might be attributed to the sequencing of the small panel of 62 genes in this study, which might fail to detect all the deletion mutations.

## Conclusion

In conclusion, targeted high-throughput sequencing can detect specific gene regions accurately and efficiently, and understanding the correlation between genomic alterations and SCLC prognosis is essential for more individualized treatment of SCLC patients. Furthermore, due to the uncharacterized function of *KIAA1211*, it is of great significance to investigate the biological function of *KIAA1211* in SCLC.

## Supplementary information

**Additional file 1: Figure S1.** Thermal map of mutation variants in SCLC.

**Additional file 2: Figure S2.** Kaplan–Meier analysis of high TMB and low TMB. a. PFS in ES-SCLC; b. OS in ES-SCLC; c. PFS in LS-SCLC; b. OS in LS-SCLCC.

**Additional file 3: Table S1.** Univariate analysis between gene mutations and PFS. **Table S2.** Univariate analysis between variants and PFS. **Table S3.** Univariate analysis between gene mutations and OS. **Table S4.** Univariate analysis between variants and OS.

## Data Availability

The datasets used and/or analyzed during the current study are available from the corresponding author on reasonable request.

## Abbreviations

ES: Extensive-stage
LS: Limited-stage
OS: Overall survival
PFS: Progression free survival
SCLC: Small cell lung cancer
TMB: Tumor mutation burden
