# Supplementary figures and images for: Prognostic impact of tumor mutation burden and the mutation in KIAA1211 in small cell lung cancer
Source: Respir Res. 2019 Nov 7;20:248. doi: 10.1186/s12931-019-1205-9 (PMC6836503; doi:10.1186/s12931-019-1205-9)

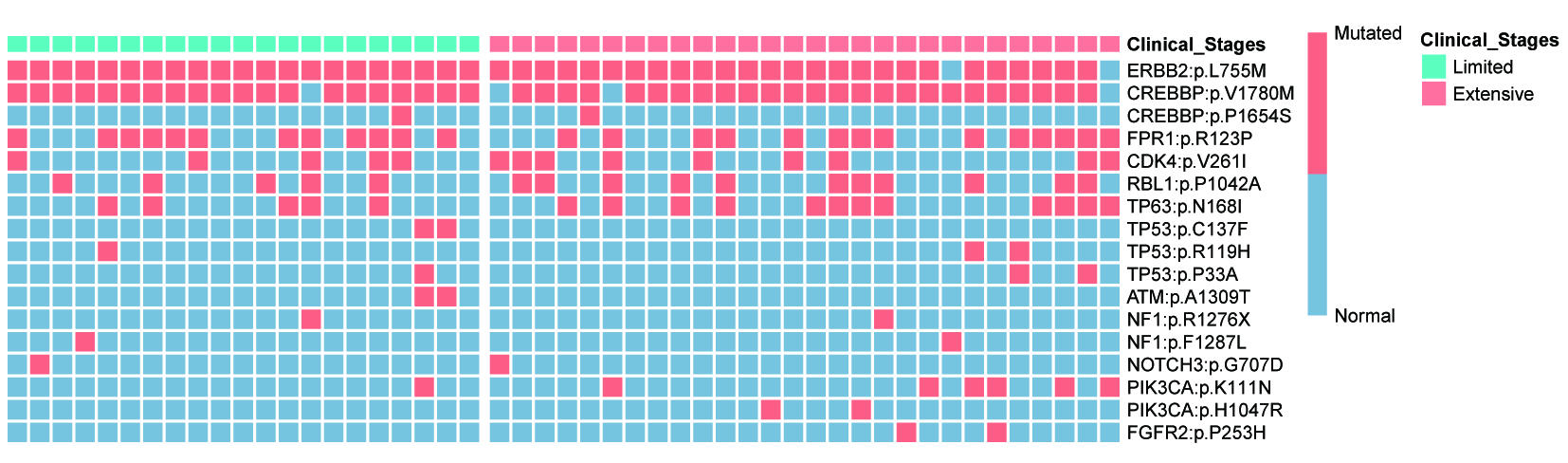

Supplement: Supplementary file 1 — Additional file 1: Figure S1. Thermal map of mutation variants in SCLC. [file 12931_2019_1205_MOESM1_ESM.tif]

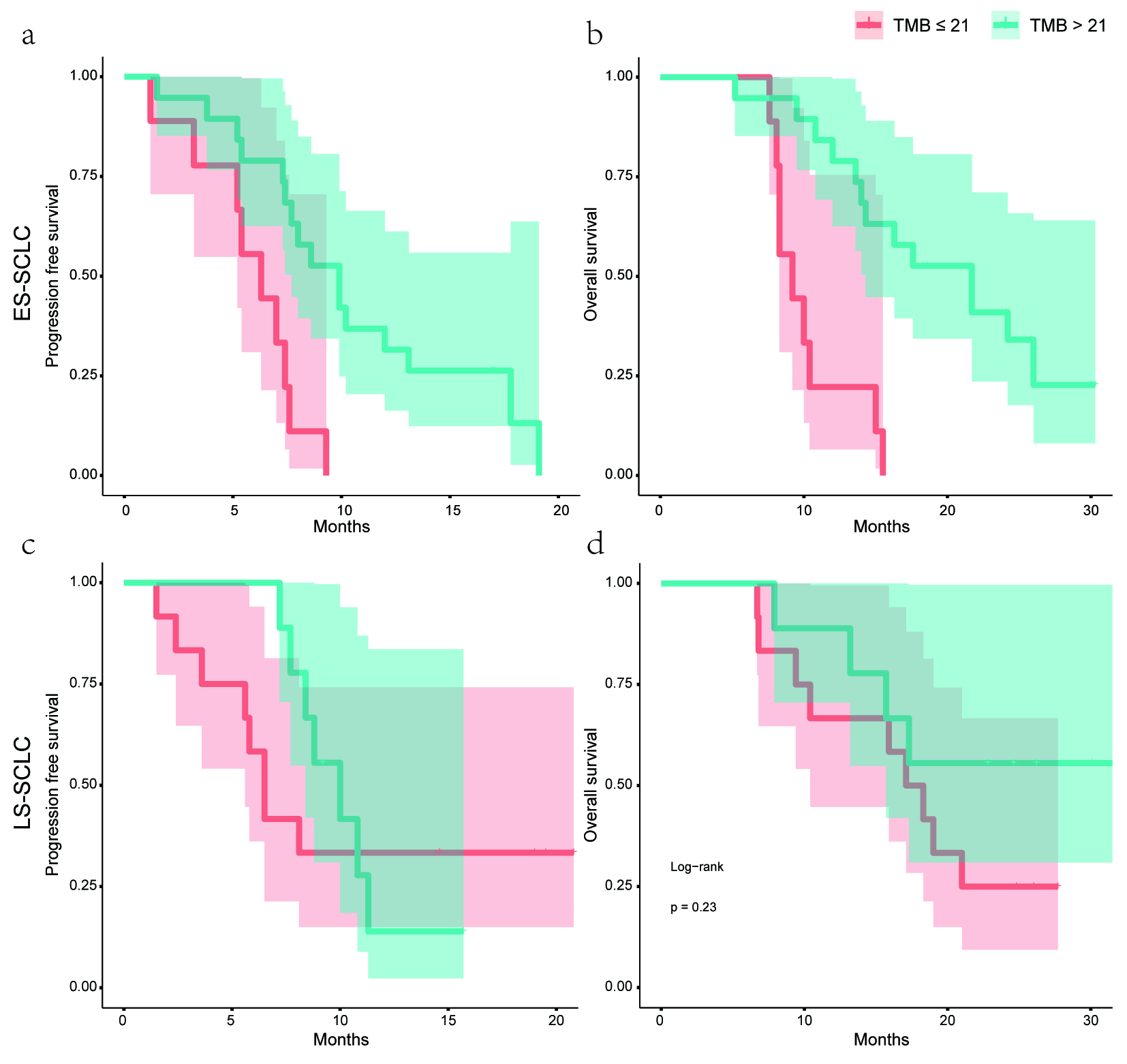

Supplement: Supplementary file 2 — Additional file 2: Figure S2. Kaplan–Meier analysis of high TMB and low TMB. a. PFS in ES-SCLC; b. OS in ES-SCLC; c. PFS in LS-SCLC; b. OS in LS-SCLCC. [file 12931_2019_1205_MOESM2_ESM.tif]
