# Supplementary material for: Prognostic impact of tumor mutation burden and the mutation in KIAA1211 in small cell lung cancer
Source: Respir Res. 2019 Nov 7;20:248. doi: 10.1186/s12931-019-1205-9 (PMC6836503; doi:10.1186/s12931-019-1205-9)
Supplement: Supplementary file 3 — Additional file 3: Table S1. Univariate analysis between gene mutations and PFS. Table S2. Univariate analysis between variants and PFS. Table S3. Univariate analysis between gene mutations and OS. Table S4. Univariate analysis between variants and OS. [file 12931_2019_1205_MOESM3_ESM.docx]

Table S1. Univariate analysis between gene mutations and PFS

| Gene | % | p value | Gene | % | p value |
| --- | --- | --- | --- | --- | --- |
| ERBB2 | 95.92 | 0.0001 | SMO | 6.12 | 0.169 |
| CREBBP | 95.92 | 0.959 | ERBB4 | 6.12 | 0.839 |
| TP53 | 77.55 | 0.127 | TP73 | 6.12 | 0.480 |
| FPR1 | 59.18 | 0.535 | BRAF | 4.08 | 0.762 |
| TP63 | 40.82 | 0.323 | NFE2L2 | 4.08 | 0.233 |
| RBL1 | 32.65 | 0.411 | RB1 | 4.08 | 0.512 |
| CDK4 | 30.61 | 0.270 | KDR | 4.08 | 0.010 |
| KIAA1211 | 20.41 | 0.089 | PTEN | 4.08 | 0.017 |
| NF1 | 18.37 | 0.496 | RBL2 | 4.08 | 0.530 |
| PIK3CA | 18.37 | 0.765 | ATM | 4.08 | 0.697 |
| NOTCH3 | 12.24 | 0.477 | EGFR | 4.08 | 0.406 |
| RASA1 | 8.16 | 0.325 | FGFR2 | 4.08 | 0.224 |
| CUL3 | 6.12 | 0.855 | KEAP1 | 4.08 | 0.727 |

Table S2. Univariate analysis between variants and PFS

| Gene | % | p value | Gene | % | p value |
| --- | --- | --- | --- | --- | --- |
| ERBB2.p.L755M | 95.92 | 0.0001 | ATM.p.A1309T | 4.08 | 0.697 |
| CREBBP.p.V1780M | 91.84 | 0.367 | CREBBP.p.P1654S | 4.08 | 0.991 |
| FPR1.p.R123P | 53.06 | 0.616 | FGFR2.p.P253H | 4.08 | 0.224 |
| CDK4.p.V261I | 28.57 | 0.535 |  |  |  |

Table S3. Univariate analysis between gene mutations and OS

| Gene | % | p value | Gene | % | p value |
| --- | --- | --- | --- | --- | --- |
| ERBB2 | 95.92 | 0.141 | SMO | 6.12 | 0.144 |
| CREBBP | 95.92 | 0.218 | ERBB4 | 6.12 | 0.299 |
| TP53 | 77.55 | 0.276 | TP73 | 6.12 | 0.919 |
| FPR1 | 59.18 | 0.728 | BRAF | 4.08 | 0.616 |
| TP63 | 40.82 | 0.900 | NFE2L2 | 4.08 | 0.470 |
| RBL1 | 32.65 | 0.269 | RB1 | 4.08 | 0.313 |
| CDK4 | 30.61 | 0.242 | KDR | 4.08 | 0.715 |
| KIAA1211 | 20.41 | 0.009 | PTEN | 4.08 | 0.193 |
| NF1 | 18.37 | 0.050 | RBL2 | 4.08 | 0.822 |
| PIK3CA | 18.37 | 0.379 | ATM | 4.08 | 0.233 |
| NOTCH3 | 12.24 | 0.702 | EGFR | 4.08 | 0.461 |
| RASA1 | 8.16 | 0.576 | FGFR2 | 4.08 | 0.630 |
| CUL3 | 6.12 | 0.274 | KEAP1 | 4.08 | 0.790 |

Table S4. Univariate analysis between variants and OS

| Gene | % | p | Gene | % | p |
| --- | --- | --- | --- | --- | --- |
| ERBB2.p.L755M | 95.92 | 0.141 | ATM.p.A1309T | 4.08 | 0.233 |
| CREBBP.p.V1780M | 91.84 | 0.677 | CREBBP.p.P1654S | 4.08 | 0.418 |
| FPR1.p.R123P | 53.06 | 0.977 | FGFR2.p.P253H | 4.08 | 0.630 |
| CDK4.p.V261I | 28.57 | 0.444 |  |  |  |
